# Recyclability of Concrete Pavement Incorporating High Volume of Fly Ash

**DOI:** 10.3390/ma8085260

**Published:** 2015-08-21

**Authors:** Isamu Yoshitake, Takeo Ishida, Sunao Fukumoto

**Affiliations:** 1 Department of Civil and Environmental Engineering, Yamaguchi University, Tokiwadai 2-16-1, Ube, Yamaguchi 755-8611, Japan; 2 Cement and Construction Materials Company, Ube Industries, Ogushi Okinoyama 1-6, Ube, Yamaguchi 755-8633, Japan; E-Mail: takeo.ishida@ube-ind.co.jp; 3 The Chugoku Electric Power Co. Inc., Komachi 4-33, Naka-ku, Hiroshima, Hiroshima 730-8701, Japan; E-Mail: 289971@pnet.energia.co.jp

**Keywords:** fly ash, recyclability, cement, concrete pavement, limestone, early strength

## Abstract

Recyclable concrete pavement was made from fly ash and crushed limestone sand and gravel as aggregates so that the concrete pavement could be recycled to raw materials for cement production. With the aim to use as much fly ash as possible for the sustainable development of society, while achieving adequate strength development, pavement concrete having a cement-replacement ratio of 40% by mass was experimentally investigated, focusing on the strength development at an early age. Limestone powder was added to improve the early strength; flexural strength at two days reached 3.5 MPa, the minimum strength for traffic service in Japan. The matured fly ash concrete made with a cement content of 200 kg/m^3^ achieved a flexural strength almost equal to that of the control concrete without fly ash. Additionally, Portland cement made from the tested fly ash concrete was tested to confirm recyclability, with the cement quality meeting the Japanese classification of ordinary Portland cement. Limestone-based recyclable fly ash concrete pavement is, thus, a preferred material in terms of sustainability.

## 1. Introduction

Asphalt concrete pavement accounts for approximately 95% of the total length of road in Japan. Cement concrete pavement accounts for less than 5% of the total length because of the concerns of construction costs and traffic noise, yet it has adequate durability and superior life-cycle cost. Most concrete pavement requires a relatively long curing term before traffic service, which negatively affects its use as a pavement material.

Fly ash collected from coal-burning power plants has remarkable properties that can improve the performance of cement concrete when added, and the powder material is, thus, often used as a supplemental binder of Portland cement in concrete (fly ash concrete). The greatest concern about fly ash concrete is the gradual strength development at an early age. Thus, when fly ash concrete is employed as a pavement material, the traffic service must be further delayed. 

Limestone powder can be added to improve strength development via the micro-filler effect and help accelerate the reaction at an early age. Bentz *et al.* [1] reported the properties of high-volume fly ash (HVFA) mixtures enhanced with the addition of limestone powder. Bentz *et al.* [2] used limestone powder with a particle size of up to five micrometers as a cement-replacement material for HVFA concrete and found that the early-age reaction was accelerated by replacing 5% of the cement volume with limestone powder. Yoshitake *et al.* [3,4] examined the tensile properties of HVFA concrete made with limestone aggregate and powder. In particular, they presented the uniaxial tensile strength and tensile Young’s modulus of the HVFA concrete. In addition, they addressed the possibility of recyclable concrete for cement production.

Fundamental properties and applications of HVFA concrete were summarized in high-performance and high-volume fly ash concrete [5]. Additionally, applications for road pavement have been introduced in the literature on HVFA concrete. Das [6] examined various properties of fresh and hardened HVFA concrete applicable for road pavement. That study investigated the design of rigid pavement using the HVFA concrete and compared the cost performance with that of conventional concrete pavement.

Naik *et al.* [7] reported the mechanical properties and durability of HVFA concrete pavement incorporating classes C and F of fly ash. In addition, Naik *et al.* [8] conducted a field study to investigate the long-term performance of concrete pavements made with a high volume of class-C and -F fly ash. They described long-term performances (ages of 7 to 14 years) and reported that long-term compressive strengths of class-F fly ash concretes were better than those of Class-C fly ash concretes. Atis and Celik [9] investigated the relations between abrasion resistance and compressive/flexural strengths of pavement concrete made with a high volume of class-F fly ash. They presented a strong linear relation between abrasion resistance and flexural strength. Atis [10] examined the strength properties of roller-compacted and workable HVFA concretes having cement replacement ratios of 50% and 70%. According to their investigation, the HVFA concrete had higher compressive and tensile strengths than normal concrete made without fly ash. They concluded that HVFA concrete is an adequate material for both structural and pavement applications. Kumar *et al.* [11] performed a laboratory test to examine compressive and flexural strengths of pavement concretes. The tested concretes were made with three water-cementitious material ratios (*w/cm* = 0.40, 0.34, 0.30) and 20%–60% cement replacement by fly ash. The test results showed that the concrete containing 40% fly ash developed the maximum strength. Rashad *et al.* [12] prepared HVFA concretes blended with silica fume and granulated blast-furnace slag, and conducted compression and abrasion tests. They reported that concrete containing 50% fly ash, 10% silica fume and 10% granulated blast-furnace slag had the maximum compressive strength among the HVFA concrete specimens. Nassar *et al.* [13] reported a field investigation of HVFA concrete pavement, including strength of concrete cores at the age of 270 days and abrasion resistance. They addressed that HVFA concrete can contribute economic construction and improve the service life of the infrastructure. 

According to the definition of HVFA concrete [5], fly ash is used as a cementitious material to replace 50% or more of the cement by mass. A concern about HVFA concrete is the strength development at an early age, while HVFA concrete has numerous advantages for structural application. Concrete pavement often requires an adequate early strength for practical use, and the strength development should thus be improved. The present study aims to develop a fly ash concrete pavement having adequate strength at an early age. The ratio of cement replacement by fly ash was designed as 40% (*FA/cm* = 0.4) by referring to previous investigations [7,11]. To improve the strength development at an early age, limestone powder was added to the fly ash concrete. Furthermore, crushed limestone was used as all fine and coarse aggregates in the concrete following previous studies [3,4]. Concrete made with limestone aggregate has a remarkably low coefficient of thermal expansion, which reduces thermal cracking in concrete pavement. It is of interest that all components of the concrete are materials that can be used in manufacturing Portland cement [14,15]. In particular, the recycling of the concrete is relatively feasible because steel-reinforcement is seldom used in most concrete pavements. The present study focuses on developing a recyclable fly ash concrete pavement having adequate early strength and confirming the recyclability for cement production. This paper presents fundamental properties of the recycled cement made from the fly ash concrete pavement.

## 2. Flexural Strength

### 2.1. Materials 

Table 1 summarizes fundamental properties of the materials used in this study. Fine and coarse aggregates used in the concrete were crushed limestone sand (2.62 g/cm^3^) and crushed limestone (2.68 g/cm^3^) so as to ensure the recyclability for cement production. To eliminate the influence of temperature on the fresh properties of concrete, all materials had been stored in a temperature-controlling room (20 °C) for 24 h or more. Powder materials in the recyclable concrete were ordinary Portland cement, limestone powder and fly ash. Table 2 gives chemical compositions of the cement and limestone powder. The limestone powder was added to improve strength development at an early age. A type-II fly ash as defined by Japanese standards [16] was used in the study; the fly ash has properties similar to those of class-F fly ash in ASTM C618 [17]. Table 3 gives the properties and chemical composition of the fly ash. A high-range water-reducing admixture (HRWRA) was used to obtain an appropriate slump (20 mm) for the pavement concrete made with a very low water-cementitious material ratio (*w/cm* = 0.33). The HRWRA (1.06 g/cm^3^) used in the study was a commercial air entraining (AE) water-reducing agent of polycarboxylic acid.

**Table 1:** Materials used in tests.

Materials	Type	Properties		
Cement (*C*)	Ordinary Portland Cement	See Table 2		
Limestone powder (*LP*)	Ground limestone powder ^a^	See Table 2		
Fly ash (*FA*)	type II	See Table 3		
**Aggregate**	**Type**	**Density**	**F.M. ^b^**	**Size**
Fine aggregate (*S*)	Crushed limestone sand	2.62 g/cm^3^	2.75	5− mm
Coarse aggregate (*G*)	Crushed limestone	2.68 g/cm^3^	N/A ^c^	20–5 mm
Admixture (*HRWRA*)	AE water-reducing agent of polycarboxylic acid			
Admixture (*AEA*)	Air entraining agent for fly ash			

^a^ UBE Tancal 200M (Ube Material Ind., Ube, Yamaguchi, Japan); ^b^ Fineness modulus; ^c^ solid content of 0.6. AE: air entraining.

**Table 2:** Physical and chemical compositions of cement and limestone powder.

Properties	Cement (*C*)	Limestone powder (*LP*)
Density	3.15 g/cm^3^	2.70 g/cm^3^
Blaine fineness	3185 cm^2^/g	5000 cm^2^/g
Setting time start-end	2 h 19 min–3 h 22 min	N/A
Comp. strength at 3 days	28.6 MPa	N/A
at 7 days	46.1 MPa	N/A
at 28 days	62.5 MPa	N/A
**Chemical Compositions**		
CaO	64.3%	55.62%
SiO_2_	20.4%	0.09%
Al_2_O_3_	5.7%	0.010%
Fe_2_O_3_	2.9%	0.013%
MgO	1.08%	0.35%
SO_3_	1.89%	0.00%
Cl^−^	0.017%	0.00%
ignition loss (ig.loss)	2.25%	43.8%

**Table 3:** Properties and chemical compositions of fly ash.

Properties	Fly ash (*FA*)
Density	2.18 g/cm^3^
Blaine fineness	3440 cm^2^/g
pH	4.6
**Chemical Compositions**	
ig.loss	2.70%
SiO_2_	58.2%
Al_2_O_3_	23.3%
Fe_2_O_3_	3.19%
CaO	0.98%
K_2_O	1.38%
MgO	0.55%
SO_3_	0.24%
Na_2_O	0.19%

### 2.2. Mixture Proportions

Concrete pavement having three mixture proportions were prepared for the experimental study (Table 4). All concretes in this investigation were made with a water-cementitious material ratio of 0.33 and had extremely low consistency, as indicated by an average slump value of 20 mm. The concrete mixture proportions were designed to achieve a specified flexural strength (4.5 MPa) at the age of seven days. It is commonly known that the limestone powder contributes strength development at an early age. The limestone powder was used as a mineral admixture for the pavement concrete. The quantity of limestone powder (50 kg/m^3^) was designed through mixing-tests of fresh concrete. The M-1 mixture was fly ash concrete incorporating limestone powder, while M-2 was fly ash concrete made with more fly ash (40 kg/m^3^) instead of limestone powder. The additional fly ash is regarded for convenience as a mineral admixture like limestone powder. The accurate cementitious material ratios are given in the parenthesis in Table 4. To achieve adequate strength development at an early age, the ratio of cement replacement by fly ash in these two concretes was designed as 0.40 by referring to previous investigations [7,11]. The M-0 concrete was a control mixture without fly ash but including limestone powder.

**Table 4:** Mixture proportions of concrete.

Mix. ID	M-0	M-1	M-2 ^d^
*FA*/*cm* ^a^	0.0	0.4	0.4 (0.47) ^d^
*w*/*cm* ^a^	0.33	0.33	0.33 (0.29) ^d^
*W*	110 kg/m^3^	110 kg/m^3^	110 kg/m^3^
*C*	334 kg/m^3^	200 kg/m^3^	200 kg/m^3^
*FA*	0 kg/m^3^	134 kg/m^3^	134 + 40 ^d^ kg/m^3^
*LP*	50 kg/m^3^	50 kg/m^3^	0 kg/m^3^
*S*	843 kg/m^3^	820 kg/m^3^	820 kg/m^3^
*G*	1062 kg/m^3^	1033 kg/m^3^	1033 kg/m^3^
*HRWRA* ^b^	4.68 kg/m^3^	4.01 kg/m^3^	3.34 kg/m^3^
*AEA* ^c^	1.23 kg/m^3^	1.20 kg/m^3^	2.00 kg/m^3^

^a^ cementitious material (cement + fly ash); ^b^ high-range water-reducing agent; ^c^ air-entraining agent ^d^ incorporating fly ash as a substitution of limestone powder.

### 2.3. Test Procedure

Thirty concrete beam specimens (100 mm × 100 mm × 400 mm) for each mixture proportion were prepared in a laboratory. All specimens were cured in a temperature-controlled room (20 °C) for 1 day and then stored in a water tank at 20 °C after demolding. To examine the strength development of the pavement concretes, four-point loading tests were conducted at ages of 1, 2, 3, 5, 7, 28, 56, 91, 182 and 364 days. The flexural strength at each test age was taken as the average strength of three beam specimens at the same test age.

### 2.4. Flexural Strength

**Figure 1:**
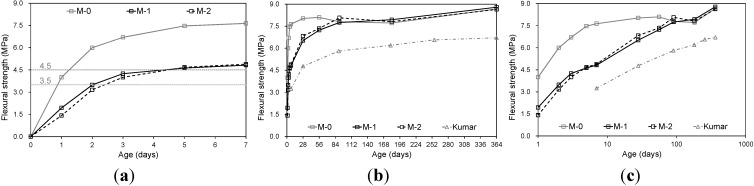
Flexural strength development; (**a**) Early age; (**b**) Linear graph; (**c**) Semi-logarithmic graph.

Figure 1a shows the flexural strength development at an early age (0–7 days). Although both fly ash concretes (M-1 and M-2) had less strength than the control concrete (M-0), the flexural strength development of M-1 was slightly greater than that of M-2 without limestone powder. The observation verified the effect of limestone powder (*i.e.*, the micro-filler effect) and the acceleration of the reaction at an early age. Concrete pavement is passable for traffic when the flexural strength is equal to or higher than 3.5 MPa [18]. The fly ash concrete M-1 achieved the required strength after two days. Both fly ash concretes had strength of approximately 4.8 MPa at an age of seven days, thus reaching the specified strength (4.5 MPa) of most concrete pavement.

Figure 1b,c shows the flexural strength development up to a concrete age of 364 days. A previous test result [11] is also shown on the graph for comparison. The referenced strength data were recorded in a test on pavement concretes incorporating class-F fly ash (*FA/cm* = 0.4, *w/cm* = 0.34). All concretes tested in the present study had higher strength than the referenced strength data [11]. The difference in strength development was probably due to the different aggregate and water contents of the concrete mixtures. Influencing factors of flexural strength development should be investigated in detail in future research. It is noted that the flexural strength of concrete made from high-early-strength Portland cement achieved the minimum strength after one day under field curing conditions [19].

Figure 1b,c shows that the flexural strengths of the fly ash concretes M-1 and M-2 gradually developed while the strength of the control concrete (M-0) hardly increased over 91 days. It is noteworthy that the flexural strengths of fly ash concretes M-1 and M-2 were almost equal to the strength of the control concrete (M-0) after one year. The ultimate strength was higher than the specified strength for common pavement concrete, while the concrete incorporated only 200 kg/m^3^ of Portland cement. The fly ash concrete tested in the present study demonstrated adequate strength development for application as pavement concrete.

## 3. Recyclability for Cement Production 

To confirm the recyclability of the pavement concrete, a Portland cement (recycled cement) was made using test specimens of the fly ash concrete. The age of the concrete specimens was half a year or more. The tested concrete was a raw material for manufacturing cement as an alternative material to limestone. The study examined the chemical and physical properties of the recycled cement.

The hardened concrete was dried in an oven (105 °C) for 24 h and crushed using a ball mill. Chemical compositions of the concrete were examined employing a Japanese standard test [20] and X-ray fluorescence analysis. The chemical compositions are summarized in Table 5.

**Table 5:** Chemical compositions of the hardened fly ash concrete.

Compositions	Percentage	Compositions	Percentage
ig.loss	39.43%	Na2O	0.04%
SiO_2_	3.25%	K_2_O	0.07%
Al_2_O_3_	1.05%	R_2_O	0.09%
Fe_2_O_3_	0.55%	TiO_2_	0.07%
CaO	50.28%	MnO	0.02%
MgO	0.47%	P_2_O_5_	0.06%
SO_3_	0.20%	Cl	0.005%

### 3.1. Cement Clinker 

A cement clinker, having components similar to those of a reference clinker [21] given in Table 6, was first produced. The fly ash concrete was used in the clinker as a primary calcium material. The materials used for the recycled cement clinker are summarized in Table 7. Fly ash and siliceous powder were added to control the amount of the silicon component in the cement clinker.

**Table 6:** Mineralogical compositions of the cement clinker and recycled cement.

Compositions	Reference Clinker ^a^	Cement-Clinker ^b^	Recycled Cement
C_3_S	56.8%	55.1%	52.9%
C_2_S	17.7%	23.1%	21.6%
C_3_A	8.9%	9.4%	8.7%
C_4_AF	8.5%	9.0%	8.9%

^a^ reference given in JCA (2007) [21]; ^b^ cement clinker made from the recycled fly-ash concrete.

**Table 7:** Materials for the cement clinker.

Materials	Unit Weight	Percentage by Mass
Fly ash concrete	848.7 kg/t	84.9%
Fly ash	67.6 kg/t	6.76%
Siliceous powder	58.0 kg/t	5.80%
Iron oxide (Fe_2_O_3_)	9.7 kg/t	0.97%
Gypsum di-hydrate	7.9 kg/t	0.79%
Sodium carbonate (Na_2_CO_3_)	2.6 kg/t	0.26%
Potassium carbonate (K_2_CO_3_)	5.4 kg/t	0.54%

The cement clinker was made with the above materials in an electric furnace. The temperature of the furnace increased from 1000 to 1200 °C over 20 min and from 1200 to 1450 °C over 10 min. The temperature was maintained at 1450 °C for 30 min and then reduced to 1350 °C. Afterward, the clinker was removed from the furnace and quenched to a room temperature of 20 °C. The chemical properties of the cement clinker made from the fly-ash concrete were then examined. The chemical compositions were examined employing the test method given in JIS R5202 [20]. The mineralogical and chemical compositions of the cement clinker are summarized in Table 6 and Table 8, respectively. The mineralogical compositions were estimated using the Bogue formulae, given as Equations (1)–(5) [22].

(1) $$C_{3}S\;=\;4.07\;\times \;\mathrm{CaO}\;-\;7.60\;\times \;\mathrm{Si}O_{2}-\;6.72\;\times \;Al_{2}O_{3}-\;1.43\;\times \;Fe_{2}O_{3}-\;2.85\;\times \;SO_{3}$$

(2) $$C_{2}S\;=\;2.87\;\times \;\mathrm{Si}O_{2}-\;0.754\;\times \;C_{3}S$$

(3) $$C_{3}A\;=\;2.65\;\times \;Al_{2}O_{3}-\;1.69\;\times \;Fe_{2}O_{3}$$

(4) $$C_{4}\mathrm{AF}\;=\;3.04\;\times \;Fe_{2}O_{3}$$

(5) $$\mathrm{CaS}O_{4}=\;1.70\;\times \;SO_{3}$$

**Table 8:** Chemical compositions of the cement clinker and recycled cement.

Compositions	Cement-Clinker	Recycled Cement	JIS R 5210 (2009) ^a^
ig.loss	0.12%	1.21%	<5.0%
SiO_2_	22.52%	21.45%	N/A
Al_2_O_3_	5.42%	5.16%	N/A
Fe_2_O_3_	2.96%	2.93%	N/A
CaO	66.04%	64.41%	N/A
MgO	0.83%	0.79%	<5.0%
SO_3_	0.67%	2.57%	<3.5%
Na_2_O	0.32%	0.31%	N/A
K_2_O	0.48%	0.52%	N/A
R_2_O	0.64%	0.65%	<0.75%
TiO_2_	0.29%	0.27%	N/A
MnO	0.05%	0.05%	N/A
P_2_O_5_	0.16%	0.15%	N/A
Cl	N/A	0.00%	<0.0035%

^a^ requirements defined in JIS R 5210. (2009) [23].

### 3.2. Chemical and Physical Properties of the Recycled Cement

The cement clinker and gypsum were mixed and ground in a ball mill to produce the recycled cement. Additionally, the chemical compositions of the recycled cement were examined, employing a Japanese standard test [20] and X-ray fluorescence analysis. From the results of the chemical tests, the mineralogical compositions of the recycled cement were calculated using the Bogue formulae [22]. The mineralogical and chemical compositions of the recycled-cement clinker are summarized in Table 6 and Table 8, respectively. It was confirmed that the chemical compositions satisfied the requirements of Portland cement defined in JIS R 5210 [23].

In addition, the study examined fundamental physical properties given in JIS R5201 [24]. Table 9 presents the test results of the recycled cement. The density of the recycled cement was slightly lower than the general value (3.15 g/cm^3^) of ordinary Portland cement in Japan. The strengths of the cement were higher than the minimum strength given in JIS R5210 [23]. It was also confirmed that the physical properties of the recycled cement meet the requirements of Portland cement, defined in JIS R5210 [23].

**Table 9:** Physical properties of the recycled cement.

Properties	Recycled Cement	JIS R 5210 (2009) ^a^
Density	3.12 g/cm^3^	N/A
Blaine fineness	3320 cm^2^/g	>2500 cm^2^/g
Residue of 90 μm sieve	0.6%	N/A
Setting time start-end	1 h 53 min–2 h 53 min	60 min–10 h
Soundness	Good	Good
Comp. strength at 3 days	32.5 MPa	>12.5 MPa
at 7 days	47.5 MPa	>22.5 MPa
at 28 days	61.5 MPa	>42.5 MPa
Flexural strength at 3 days	6.7 MPa	N/A
at 7 days	8.0 MPa	N/A
at 28 days	9.0 MPa	N/A
Flow value	208 mm	N/A

^a^ requirements defined in JIS R 5210. (2009) [23].

## 4. Conclusions

The present study investigated recyclable pavement concrete made with a cement-replacement ratio of 40% by mass. The early-age strength is the most concern relating to the practical use of fly ash concrete, and the study thus aimed to develop a concrete pavement having adequate early strength. Limestone aggregate was used to ensure recyclability for cement production, and limestone powder was added to the concrete of very low water-cementitious material ratio (*w/cm* = 0.33) to improve strength development at an early age. The study examined the development of flexural strength up to age of 364 days, and examined physical and chemical properties of the recycled cement to confirm recyclability. The conclusions drawn from the results of the study are as follows.

- The fly ash concrete with limestone powder achieved the minimum strength (3.5 MPa) for passable pavement at an age of two days, and the specified strength (4.5 MPa) of most concrete pavement at an age of seven days. Furthermore, the matured fly ash concrete achieved a strength almost equal to that of the control concrete without fly ash despite the concrete being made with a unit cement weight of 200 kg/m^3^.
- The fly ash concrete was used in cement manufacturing as a primary calcium material. Fundamental examinations revealed that the chemical and physical properties of the recycled cement meet the quality of ordinary Portland cement defined in Japanese industrial standards. It is concluded that the recyclable fly ash concrete pavement is a preferred material in terms of sustainability.

Although the recycled cement made in the test furnace has shown promising test results, further investigations are necessary to confirm the practical use of the cement, such as production process and cost performance.
